# A hypomorphic *SRD5A2* haplotype with a potential founder effect: composed of common variants in individuals with 5α-reductase type 2 deficiency from South China

**DOI:** 10.1186/s13293-026-00847-3

**Published:** 2026-02-11

**Authors:** Xiaoyun Lei, Xu Zhou, Zifeng Cheng, Sen Zhao, Chunrong Gui, Yunting Ma, Meizhen Shi, Xianda Wei, Bobo Xie, Xin Fan, Shaoke Chen, Qiuxing Tao, Yuna Su, Dejian Yuan, Baoheng Gui

**Affiliations:** 1 Center for Medical Genetics and Genomics, The Second Affiliated Hospital of Guangxi Medical University, No. 166, Daxuedong Road, Xixiangtang District, Nanning, 530007 Guangxi Zhuang Autonomous Region China; 2 The Guangxi Health Commission Key Laboratory of Medical Genetics and Genomics, The Second Affiliated Hospital of Guangxi Medical University, No. 166, Daxuedong Road, Xixiangtang District, Nanning, 530007 Guangxi Zhuang Autonomous Region China; 3 The Second School of Medicine, Guangxi Medical University, No. 166, Daxuedong Road, Xixiangtang District, Nanning, 530007 Guangxi Zhuang Autonomous Region China; 4 Peking Union Medical College Hospital, Chinese Academy of Medical Sciences & Peking Union Medical College, No. 1 Shuaifuyuan, Beijing, 100730 China; 5 Department of Pediatrics, The Second Affiliated Hospital of Guangxi Medical University, No. 166, Daxuedong Road, Xixiangtang District, Nanning, 530007 Guangxi Zhuang Autonomous Region China; 6 Department of Medical Genetics, Liuzhou Hospital of Guangzhou Women and Children’s Medical Center, No. 50, Boyuan Avenue, Yufeng District, Liuzhou, 545616 Guangxi Zhuang Autonomous Region China

**Keywords:** *SRD5A2*, Hypomorphic haplotype, Founder effect, 5α-Reductase type 2 deficiency, Disorders of sex development, South China

## Abstract

**Background:**

Disorders of sex development (DSDs) exhibit high genetic and phenotypic heterogeneity, and genotype–phenotype correlations are not fully understood. 5α-Reductase type 2 (5α-RD2) deficiency, a common form of DSD, is caused by *SRD5A2* inactivation. This study investigated the role of *SRD5A2* haplotypes in DSD, focusing on their corresponding phenotypes, structural changes and impacts on enzyme activity.

**Methods:**

This study enrolled 216 individuals with DSD who underwent genetic analysis and 2,794 controls. Linkage disequilibrium analysis was performed in individuals with 5α-RD2 deficiency to identify *SRD5A2* haplotypes, and haplotype frequencies were analysed across cohorts. The clinical manifestations of individuals with different *SRD5A2* haplotypes were characterized. Structural predictions were employed to investigate the impacts of haplotypes on the 5α-RD2 structure and interactions with ligands. Functionally, kinetic assays were conducted to validate the effects of different haplotypes on enzyme activity.

**Results:**

A *SRD5A2* haplotype composed of c.265C > G and c.680G > A (Hap3: G-A) was identified, and the haplotype frequency was 64.71% in individuals with 5α-RD2 deficiency, 2.59% and 1.22% in non-5α-RD2 deficiency DSD cases without or with known DSD-related gene variants, respectively, and 1.57% in in-house controls. Globally, Hap3: G-A was enriched in southern Chinese individuals and showed high population differentiation, indicating a potential founder effect of the haplotype. The majority of homozygotes of Hap3: G-A presented microphallus, and nearly half of them manifested isolated microphallus. Structurally, Hap3: G-A was predicted to result in an increase in the solvent-accessible surface area (10.72 Å^2^), a redistribution of hydrogen bonds within 5α-RD2, and a loss of key hydrogen bonds with NADPH. Functionally, kinetic assays showed that the catalytic efficiency of the enzyme encoded by Hap3: G-A was between that of Hap1: G-G and that of Hap2: C-A.

**Conclusions:**

Hap3: G-A, which is prevalent in individuals with 5α-RD2 deficiency, suggests a potential founder effect. Structurally, compared with other haplotypes, Hap3: G-A seems to have a combined effect on the structure and interaction of 5α-RD2, rather than have merely additive effects of its constituent variants. Functionally, kinetic assays suggested a hypomorphic effect of Hap3: G-A. These findings provide valuable insights for understanding genotype–phenotype correlations, genetic counselling, early intervention and clinical management of individuals with 5α-RD2 deficiency or even other DSDs.

**Supplementary Information:**

The online version contains supplementary material available at 10.1186/s13293-026-00847-3.

## Highlights

- On the basis of 216 DSD individuals and 2,794 controls, a novel *SRD5A2* haplotype (Hap3: G-A), which is composed of common variants and is especially prevalent in DSD patients with 5α-RD2 deficiency, was identified, indicating the potential founder effect of Hap3: G-A.
- The majority of 5α-RD2 deficiency individuals with homozygous Hap3: G-A presented microphallus.
- Three-dimensional structure and model construction predicted that Hap3: G-A resulted in an increase in the solvent-accessible surface area, redistribution of hydrogen bonds within 5α-RD2, and loss of key hydrogen bonds with NADPH.
- Functionally, kinetic assays showed that the catalytic efficiency of the enzyme encoded by Hap3: G-A was between those of Hap1: G-G and Hap2: C-A, suggesting a hypomorphic effect of this haplotype.

**Supplementary Information:**

The online version contains supplementary material available at 10.1186/s13293-026-00847-3.

## Plain Language Summary

Individuals with disorders of sex development (DSDs) present variable genotypes and phenotypes, and the genotype–phenotype correlation remains poorly understood. 5α-Reductase type 2 (5α-RD2) deficiency, a common form of DSD, is caused by *SRD5A2* inactivation. In this study, genetic testing and analysis were performed in 216 individuals with DSD and 2,794 non-DSD controls. A novel *SRD5A2* haplotype (Hap3: G-A) was identified, and the haplotype frequency was 64.71% in individuals with 5α-RD2 deficiency, 2.59% and 1.22% in non-5α-RD2 deficiency DSD cases without or with known DSD-related gene variants, respectively, and 1.57% in in-house controls. Globally, Hap3: G-A was enriched in southern Chinese individuals and showed high population differentiation, indicating a potential founder effect of the haplotype. Analyzing the phenotype spectrum of individuals with 5α-RD2 deficiency, we found that the majority of homozygotes of Hap3: G-A presented microphallus, and nearly half of them manifested isolated microphallus. Structurally, Hap3: G-A was predicted to result in an increase in the solvent-accessible surface area, the redistribution of hydrogen bonds within 5α-RD2, and the loss of key hydrogen bonds with NADPH. Compared with other haplotypes, Hap3: G-A seemed to have a combined effect on the structure and interaction of 5α-RD2, rather than simply additive effects of its constituent variants. Functionally, kinetic assays showed that the catalytic efficiency of the enzyme encoded by Hap3: G-A was between that of Hap1: G-G and that of Hap2: C-A, suggesting a hypomorphic effect of this haplotype. These findings provide valuable insights for understanding genotype–phenotype correlations, genetic counselling, early intervention and clinical management of individuals with 5α-RD2 deficiency or even other DSDs.

**Supplementary Information:**

The online version contains supplementary material available at 10.1186/s13293-026-00847-3.

## Introduction

Rare congenital conditions characterized by incongruence among chromosomal, gonadal, and phenotypic sex characteristics are medically classified as differences (or disorders) of sex development (DSDs) [1–3]. According to the consensus, DSDs are classified into three groups: 46, XY DSD; 46, XX DSD; and sex chromosomal DSD [4]. Among these, 46, XY DSD is considered the most complex type, affecting approximately 1 in 6,000 live births [4, 5]. The genotype–phenotype correlation among DSD individuals has still not been fully elucidated, even for those carrying the same variant [6]. 5α-Reductase type 2 (5α-RD2) deficiency (OMIM: #264600), a rare autosomal recessive disorder, a relatively common type of 46, XY DSD, is caused by a lack or deficiency of 5α-RD2 [7]. Since the first reported 5α-RD2 deficiency individuals [8], an increasing number of cases with many types of features ranging from nearly female external genitalia to undervirilized male genitalia, including penile hypospadias and isolated microphallus have been described [9]. Even siblings from the same family who carry the same variant present different gender identities [10].

5α-RD2 plays a crucial role in catalyzing the conversion of testosterone to the more potent dihydrotestosterone (DHT) via the use of NADPH as a hydrogen donor during the hormone biosynthetic process [8, 11]. 5α-RD2 is highly expressed in androgen-sensitive tissues, including the prostate, external genitalia, epididymis, and foreskin, during fetal development [12]. Thus, 5α-RD2 is important for regulating the differentiation and formation of male external genitalia by maintaining an appropriate DHT level. During embryonic development, in the absence of DHT stimulation, 46, XY DSD can present with a wide spectrum of phenotypes, ranging from female-typical external genitalia to clitoromegaly or microphallus, anatomic anomalies such as cloacal exstrophy, hypospadias, and cryptorchidism [13–15].

5α-RD2 is encoded by the *SRD5A2* gene (OMIM: #607306). Different variants of *SRD5A2* result in variable degrees of enzyme activity and are found in patients with diverse clinical manifestations. For example, the frameshift variant c.655delT led to a complete loss of enzymatic activity, and this variant was correlated with penoscrotal hypospadias, a clitoris-like phallus [16]. Other types of variants, such as missense variants, result in a partial reduction in 5α-RD2 enzymatic activity. For example, the variant *SRD5A2* c.680G > A p.R227Q can largely reduce enzyme activity to 3.2% and is found in individuals with clitoromegaly or clitoris penis as well as hypospadias [17], which are considered pathogenic or likely pathogenic [18]. However, other missense variants, such as *SRD5A2* c.265C > G p.L89V, reportedly mildly decrease the activity of the enzyme to 80–92% [17, 19–21], and c.265C > G is common in healthy individuals according to the Genome Aggregation Database (gnomAD) [22]. This variant is often considered benign [17, 18]. The pathogenesis of *SRD5A2* missense variants remains controversial, primarily because the correlation between the threshold of enzyme activity reduction and phenotypes remains inconclusive [23], highlighting the complex association between genotype and phenotype and suggesting that other factors possibly play a role in DSD pathogenesis.

Our previous multicenter study revealed that the c.680G > A variant is a hotspot site in the Chinese population, especially in South China, indicating a potential founder gene effect of *SRD5A2* [24]. Interestingly, we found that some individuals with 5α-RD2 deficiency harboring the c.680G > A variant also carried c.265C > G [24]. This suggested that such variants might be in linkage and compose a haplotype, further implying a possible founder effect of *SRD5A2*. Moreover, it remains unclear whether the effect of a haplotype is equivalent to the accumulated or overlapping effects of two or more linked variants. To the best of our knowledge, few studies have examined the founder effect of *SRD5A2* from the perspective of haplotype analysis, especially analysis based on c.680G > A [10, 25]. Some haplotypes are hypomorphic, which results in partial loss of gene function but not complete null; for example, a common hypomorphic haplotype of *TBX6* is found in vertebral malformation, defined by the nonreference alleles of three common single-nucleotide variants (SNVs), which cause only a moderate reduction in protein function [26]. Therefore, exploring hypomorphic haplotypes may provide novel insights into the intricate genotype–phenotype relationships for diseases with high heterogeneity in genetic factors and clinical manifestations.

In this study, linkage disequilibrium (LD) analysis focusing on *SRD5A2* variants was performed, and haplotypes composed of c.265C > G and c.680G > A were identified. The frequency of the specific haplotype was calculated in individuals with 5α-RD2 deficiency or non-5α-RD2 deficiency DSD and further compared with controls from in-house and public cohorts. The clinical manifestations of individuals with different haplotypes were analysed. Additionally, to elucidate the molecular mechanisms of the haplotype in 5α-RD2 deficiency, we sought to predict the structural effects of the haplotype on enzyme stability and binding with NADPH and testosterone, and further functional assays were performed to validate the influence on enzyme activity.

## Methods

### Participant recruitment and phenotype evaluation

Participants were recruited from the genetic and endocrine metabolism specialist outpatient departments of pediatrics at the Second Affiliated Hospital of Guangxi Medical University and Liuzhou Hospital of Guangzhou Women and Children’s Medical Center from 2015 to 2024. They were referred to pediatric endocrinologists for clinical evaluation according to the criteria of the European Society for Pediatric Endocrinology and Lawson Wilkins Pediatric Endocrine Society, followed by molecular genetic analysis at their respective hospitals. Individuals assigned male at birth who exhibited external genital features indicative of incomplete virilization, including hypospadias, microphallus, and cryptorchidism, were enrolled in the study. Additionally, phenotypically female individuals with a 46,XY karyotype were also included (Supplementary Tables S1 and S3). The study controls contained two subsets: the in-house controls consisted of 290 unrelated individuals without apparent genital malformations (Supplementary Table S5), and the public controls comprised 2,504 unrelated healthy individuals from the 1000 Genomes Project (Supplementary Table S7) [27]. The enrolled patients and in-house controls were mainly from South China. The project was approved by the Institutional Medical Ethics Review Board of the Second Affiliated Hospital of Guangxi Medical University (2021-KY-0014). Informed consent was obtained from the parents of the participants.

### Genetic analysis by DSD-panel or whole-exome sequencing

The genomic DNA extracted from the peripheral blood was used for library preparation, where different capture kits were utilized depending on the target regions. For a subset of patients in the 5α-RD2 deficiency cohort, molecular genetic testing was based on next-generation sequencing (NGS) focused on a comprehensive panel containing DSD-related genes (Supplementary Table S8). For this DSD-related gene panel, the SureSelect Custom Library Kit (Agilent, Santa Clara, CA, USA) was used. For the remaining individuals with DSD, including the majority of individuals with 5α-RD2 deficiency et al., and other in-house controls, whole-exome sequencing (WES) was performed. For WES, the SureSelect Human All Exon V6 Library Kit (Agilent) or IDT xGen Exome Research Panel V2 probes were used. The prepared libraries were sequenced at an average depth of more than 100 × on high-throughput NGS platforms, such as the Illumina NovaSeq 6000 series, according to the manufacturer’s instructions.

### Sequencing data analysis and haplotype calling

The raw sequencing reads were subjected to quality control via NGSQCToolkit, and poor-quality reads were removed, followed by alignment to the reference human genome sequence via BWA software [28, 29]. After excluding the duplicated reads and performing statistical analysis on the remaining reads, variants were called via GATK software [30]. The called variants were annotated based on public databases for variant records and population frequencies, such as gnomAD [22]. Candidate variants were further confirmed by Sanger sequencing.

To identify SNVs in LD with c.680G > A, only variants located in the *SRD5A2* region and 2 Mb upstream or downstream were included. For the probands in the 5α-RD2 deficiency cohort, pairwise LD measures (D’ and R^2^), i.e., the estimated haplotype frequencies and those expected under linkage equilibrium, were statistically inferred via the expectation maximization (EM) algorithm using PLINK software (https://zzz.bwh.harvard.edu/plink/) [31]. The c.680G > A and its flanking linked SNVs were used to construct different haplotypes. For individuals homozygous for specific loci, haplotypes can be directly determined. For those remaining heterozygous for these loci, haplotypes can be assigned by pedigree analysis if their family members’ genotypes are available and informative. To evaluate the population differentiation of the assigned haplotype, *F*_ST_ values across populations were calculated via VCFtools version 0.1.17.

### Three-dimensional (3D) structure prediction and model construction for 5α-RD2 with NADPH and testosterone

To investigate the effects of *SRD5A2* haplotypes on the enzyme itself and its interactions with the cofactor NADPH and the substrate testosterone, a 5α-RD2 structure model was built based on the resolved crystal structure (Protein Data Bank ID: 7BW1) [7]. Since the 7BW1 structure contains unresolved residues (N-terminus 1–4 and loop L1 residues 39–43) and lacks the corresponding ligand model, the full-length structure was predicted and completed via Chai-1 [32]. Restrained optimization was then performed to construct four ternary models containing NADPH and testosterone, each in combination with 5α-RD2 encoded by wild-type (WT) or mutant haplotypes. During construction, residue 227 of 5α-RD2 was restrained within 5 Å of NADPH (according to the ligand interaction sites in the 7BW1 structure), and residue 114 of 5α-RD2 was restrained within 5 Å of testosterone to compensate for the missing parts and to ensure that the structure was near the active site. To analyse the solvent-accessible surface area (SASA), interaction networks, and key interacting residues of the models, PyMOL software was used [33]. The binding modes and active site changes caused by haplotypes were compared to provide a structural basis, which is helpful in predicting enzyme catalytic activity under WT and mutant haplotypes.

### Cell culture and *SRD5A2* plasmids transfection

HEK293T cells were cultured in 293 T cell-specific medium (CM-0005, Procell, Wuhan, China) at 37 °C with 5% CO_2_. The WT *SRD5A2* plasmid was constructed through linearized vectors obtained by digestion with restriction endonucleases. The resulting PCR amplimers were digested with XbaI and BsrGI and then ligated into the plasmid 3Flag-EGFP (supplied by Shanghai Genechem Co., Ltd.). The resulting plasmids were identified via Sanger sequencing (forward primer: 5′-CGCAAATGGGCGGTAGGCGTG-3′; reverse primer: 5′-TTATTAGGAAAGGACAGTGGG-3′). The plasmid was subsequently transformed into *E. coli* and further validated via Sanger sequencing. The cells were transfected with Lipofectamine 2000 Transfection Reagent (11668–019; Invitrogen, California, USA) according to the manufacturer’s instructions. The cells were collected after 48 h of transfection, and western blotting (WB) was performed to validate 5α-RD2 overexpression via primary antibodies (TA-05 and TA-08, ZSGB-BIO, Beijing, China) and secondary antibodies (ZB-2305, ZSGB-BIO, Beijing, China).

### Kinetic assays of 5α-RD2 by liquid chromatography-tandem mass spectrometry (LC–MS/MS)

HEK293T cells transfected with control vector GFP, *SRD5A2* WT or haplotypes were cultured with media supplemented with 10 μM testosterone and various concentrations of NADPH (0.0625–2 μM) (CAS: 58–22-0, N8100; Solarbio, Beijing, China) or 500 μM NADPH and various concentrations of testosterone (0.25–8.0 μM) (CAS: 2646–71-1, IT0110; Solarbio, Beijing, China). After 4 h, the medium was collected, and steroid hormones were extracted with ethyl acetate. Analytes were subjected to protein precipitation combined with liquid extraction for LC–MS/MS machine (ACQUITY UPLC I-Class IVD/Xevo TQ-S IVD System, Waters Corporation, Milford, USA) analysis, with mobile phase A containing ammonium fluoride and phase B being alcohol methanol, which was quantified according to the peak steroid hormone area. Protein concentrations were determined via BCA assays (P0012S, Beyotime, Shanghai, China). The rate of DHT production (pmol/mg protein/h) was calculated. The enzyme kinetics was analysed via nonlinear regression (curve fit) based on the Michaelis–Menten model in GraphPad Prism version 10.1.2. This analysis generated the maximum velocity (Vmax) values of the enzymes encoded by the specific haplotype.

### Statistical analysis

Statistical analysis was performed via SPSS version 27 (IBM). Two-sided Fisher’s exact test was used to evaluate differences in haplotype frequencies among cohorts, including individuals with 5α-RD2 deficiency, individuals with non-5α-RD2 deficiency DSD, in-house and public controls. For all subsequent pairwise comparisons, the Bonferroni correction was applied. Statistical significance was defined as p < 0.05. GraphPad Prism version 10.1.2 was used for visualization.

## Results

### Enrollment of the case and control cohorts

In total, 39 individuals with 5α-RD2 deficiency were enrolled. Among them, 26 cases underwent WES, while the remaining cases underwent panel sequencing targeting DSD-related genes (Supplementary Table S1). A total of 177 individuals with non-5α-RD2 deficiency DSD were subsequently recruited (Supplementary Table S3). For comparison, we collected 290 in-house controls without DSD-related phenotypes, primarily from South China (Supplementary Table S5), as well as public controls of 2,504 individuals who claimed themselves to be healthy from diverse ethnicities and geographical regions, including southern Han Chinese and Dai individuals in Xishuangbanna, China (Supplementary Table S7) [27].

### Assignment of *SRD5A2* haplotypes in the case and control cohorts

**Fig. 1:**
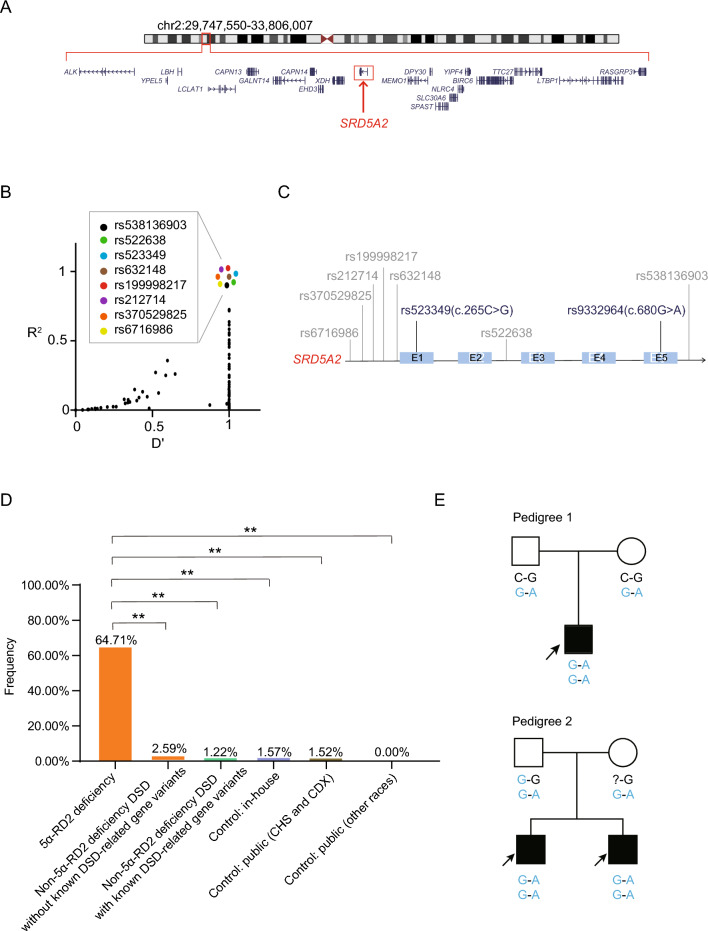
The *SRD5A2* haplotype is especially prevalent in individuals with 5α-RD2 deficiency. **A** Location information of the *SRD5A2* gene. **B** Linkage of rs9332964 (*SRD5A2* c.680G > A) with other loci. **C**. Position information of loci associated with rs9332964 on *SRD5A2*. **D** *SRD5A2* Hap3: G-A is composed of nonreference alleles of two single-nucleotide variants (SNVs), rs523349 and rs9332964, and this haplotype’s frequency profile in the case and control cohorts. All P values were calculated via two-sided Fisher’s exact tests. ** denotes p < 0.001. **E** Two pedigrees with *SRD5A2* Hap3: G-A. Squares denote male members, circles represent female pedigree members, solid symbols represent members of individuals with 5α-RD2 deficiency, and open symbols represent unaffected members; the probands are indicated by black arrows. The nonreference alleles of rs523349 and rs9332964 are shown in blue. The question mark denotes uncertainty

Considering that WES is more comprehensive than the DSD panel in calling variants, only the WES dataset of individuals with 5α-RD2 deficiency was used to construct haplotypes. The LD pattern between the SNV rs9332964 (c.680G > A) and all the flanking SNVs (located in the 2 Mb range upstream and downstream of the *SRD5A2* gene) was analysed (Fig. 1A). Many loci were found to be in LD with rs9332964, and eight loci, such as rs538136903 and rs522638, had the highest correlation (D’ = 1, R^2^ = 1) (Fig. 1B). Some loci were located upstream of *SRD5A2*, some downstream, and some inside the gene, which were located in exons or introns (Fig. 1C). Since gene function is influenced mainly by exons, we selected rs523349 (c.265C > G) for rs9332964 (c.680G > A)-based haplotype assignment (Fig. 1C). On the basis of reference and altered allele combinations of the two loci, there were WT haplotype C-G and three alternative haplotypes: haplotype G-G was defined as Hap1, C-A as Hap2, and G-A as Hap3.

For individuals with 5α-RD2 deficiency (Supplementary Table S1), five out of 39 were heterozygous at both *SRD5A2* c.265C > G and c.680G > A; for these five individuals, their parental genotype information was unavailable or incomplete; thus, haplotype status could not be clearly determined (Supplementary Table S2). For individuals with non-5α-RD2 deficiency DSD (Supplementary Table S3), one out of 177 was heterozygous at both sites, and the parental genotype was unavailable for haplotyping (Supplementary Table S4). For in-house controls (Supplementary Table S5), ten out of 290 individuals were heterozygous at both sites; among the ten individuals, six obtained haplotyping results based on their family members’ genotypes, but the remaining four failed to be haplotyped (Supplementary Table S6). Among the public controls, one out of 2,504 was heterozygous at both loci and was unable to be assigned haplotypes due to missing information of the family genotypes (Supplementary Table S7).

### *SRD5A2* Hap3: G-A may constitute a founder effect of the population in South China

To investigate the distribution of haplotypes, their frequencies in the cases and controls were calculated. Hap3: G-A in individuals with 5α-RD2 deficiency was the highest at 64.71%, which was significantly different from that in the other case and control cohorts (Fig. 1D). Interestingly, the frequency of Hap3: G-A in non-5α-RD2 deficiency DSD cases without known DSD-related gene variants was 2.59%, which was higher than that in non-5α-RD2 deficiency DSD cases with known DSD-related gene variants, as well as other control cohorts (Fig. 1D). Specifically, seven non-5α-RD2 deficiency DSD cases without known DSD-related gene variants, harboring Hap3: G-A, were identified (Table 1). We further analysed five pedigrees with 5α-RD2 deficiency, in which the parental genotypes were available and informative, and found that Hap3: G-A was inherited rather than de novo, for example, pedigrees 1 and 2, as shown in Fig. 1E.

**Table 1:** Seven non-5α-RD2 deficiency DSD cases without known DSD-related gene variants were carriers of hap3: G-A

ID	Gender	Age (Year)	Phenotype	Haplotypes
DSD-2-14	Male	1.6	Microphallus	-Hap1- -Hap3-
DSD-2-86	Male	2	Microphallus	-Hap1- -Hap3-
DSD-2-123	Male	0.3	Microphallus	-Hap1- -Hap3-
DSD-2-133	Male	10	Microphallus	-Hap1- -Hap3-
DSD-2-169	Male	10.8	Concealed penis and testicular calculi	-Hap1- -Hap3-
DSD-2-176	Male	11	Microphallus	-Hap1- -Hap3-
DSD-2-177	Male	13	Microphallus	-Hap1- -Hap3-

**Fig. 2:**
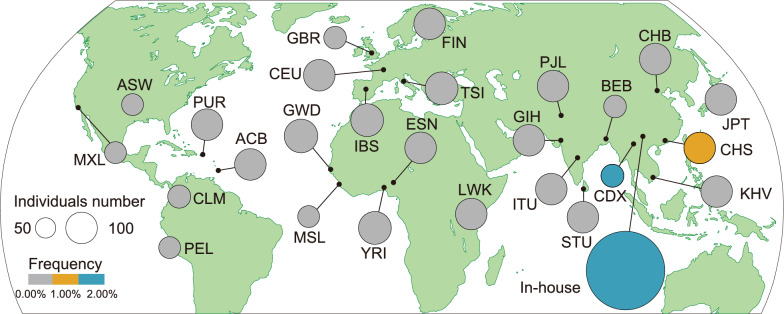
The distribution of *SRD5A2* Hap3: G-A worldwide. The public controls from South China consisted of southern Han Chinese (CHS, n = 105) and Dai individuals in Xishuangbanna, China (CDX, n = 92). The size of the circles denotes individuals’ numbers of specific populations, and the color denotes the frequency of Hap3: G-A

The distribution of Hap3: G-A in populations worldwide was further explored. In general, there are five types of populations: East Asian populations (including Han Chinese in Beijing (CHB), etc.), European populations (including Toscani in Italia (TSI), etc.), African populations (including Yoruba in Ibadan, Nigeria (YRI), etc.), and mixed-race Americans (including Mexican Ancestry from Los Angeles, USA (MXL), etc.). The population frequency of Hap3: G-A was 1.57% in our South China in-house group, 1.63% in Chinese Dai individuals in Xishuangbanna (CDX), and 1.43% in Southern Han Chinese (CHS) individuals but was absent in other ethnic groups. This finding suggests that Hap3: G-A may constitute a founder effect in southern Chinese individuals (Fig. 2). To further evaluate the population differentiation of Hap3: G-A, c.265C > G was chosen as the leading allele for the haplotype because it is common in the general population. Specifically, we calculated *F*_ST_ values for c.265C > G across populations (Table 2), which exhibited relatively high population differentiation between southern Chinese individuals (CDX and CHS) and populations of African, European or American ancestry but relatively low population differentiation between southern Chinese individuals and populations of East or South Asian ancestry (Table 2)*.*

**Table 2:** *F*_ST_ values of southern Chinese (CHS and CDX) individuals of East Asian ancestry compared with those of other populations from the 1000 genomes project

Races	Class	The *F*_ST_ values of c.265C > G	Population differentiation
CHB	East Asian ancestry	0.00	*F*_ST_ = 0: extremely low
KHV	East Asian ancestry	0.00	
BEB	South Asian ancestry	0.01	0 < *F*_ST_ < 0.05: low
PJL	South Asian ancestry	0.04	
STU	South Asian ancestry	0.04	
PEL	American ancestry	0.04	
MXL	American ancestry	0.05	0.05 < *F*_ST_ < 0.15: median
JPT	East Asian ancestry	0.05	
ITU	South Asian ancestry	0.06	
GIH	South Asian ancestry	0.08	
TSI	European ancestry	0.09	
PUR	American ancestry	0.11	
ASW	African ancestry	0.13	
CEU	European ancestry	0.15	
GBR	European ancestry	0.15	0.15 < *F*_ST_ < 0.25: high
FIN	European ancestry	0.16	
LWK	African ancestry	0.16	
ESN	African ancestry	0.16	
CLM	American ancestry	0.17	
ACB	African ancestry	0.17	
YRI	African ancestry	0.20	
IBS	European ancestry	0.20	
GWD	African ancestry	0.26	0.25 < *F*_ST_: extremely high
MSL	African ancestry	0.27	

### Phenotypic analysis of individuals with different *SRD5A2* haplotypes

**Fig. 3:**
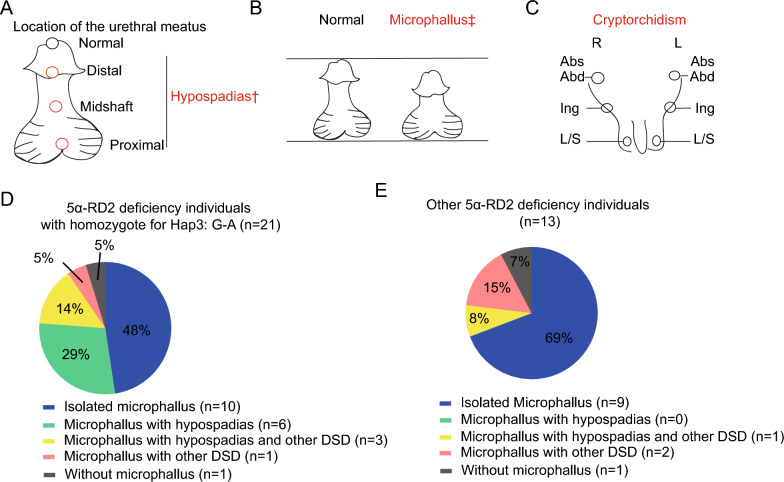
Phenotypic analysis of individuals homozygous for *SRD5A2* Hap3: G-A. **A–C** Schematic diagram of hypospadias, microphallus, and the cryptorchidic phenotype. **D** Clinical manifestations in individuals with 5α-RD2 deficiency and homozygosity for Hap3: G-A (n = 21). The pie charts show the constituent ratios of different clinical phenotypes in each subcategory. † denotes different locations of the urethral meatus [24, 45]. ‡ denotes microphallus, which was defined as a stretched penile length < 2.5 SDs below the mean for normal same-age individuals [24, 45]

Individuals with DSD presented mainly hypospadias, microphallus, and cryptorchidism (Fig. 3A–C). Since 5α-RD2 deficiency is autosomal recessive, only individuals homozygous for specific haplotypes were included to further characterize the phenotype spectrum. Among the 21 homozygotes of Hap3: G-A, 47.62% (10/21) presented isolated microphallus, followed by microphallus with hypospadias (28.57%, 6/21), microphallus with hypospadias and other DSD features (14.28%, 3/21), microphallus with other DSD features (4.76%, 1/21), and other manifestations (4.76%, 1/21) (Fig. 3D). Among the six homozygotes of Hap2: C-A, 83.33% presented isolated microphallus, and the remaining individual in this group presented microphallus with other DSD features (Supplementary Table S1). Interestingly, seven other non-5α-RD2 deficiency DSD cases without known DSD-related gene variants were carriers of Hap3: G-A, six of which presented isolated microphallus (Table 1).

### Analysis of intramolecular interactions within 5α-RD2

**Fig. 4:**
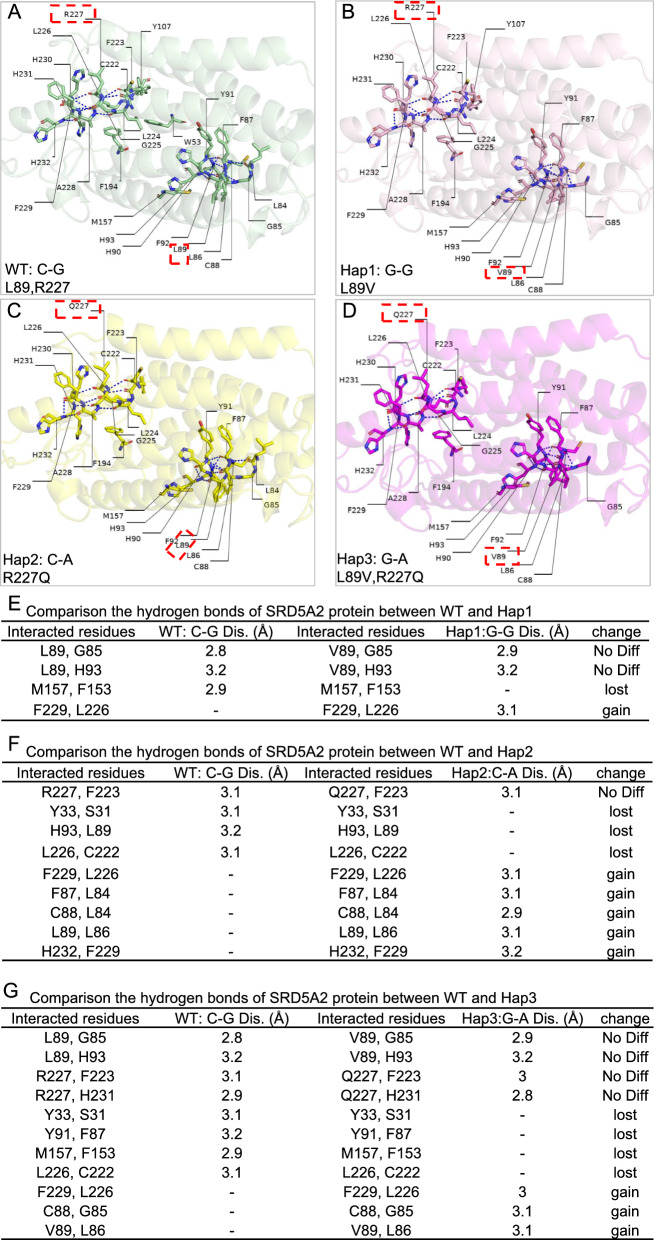
Stability analysis of 5α-RD2 based on hydrogen bonds. **A**–**D** Visualization of hydrogen bonds within a 5 Å radius around amino acid positions 89 and 227 of the wild-type (WT): C-G (**A**), Hap1: G-G (**B**), Hap2: C-A (**C**), and Hap3: G-A (**D**) derived 5α-RD2, respectively. Residues 89 and 227 are highlighted with a red box. Hydrogen bonds are represented as blue dashed lines. **E**–**G** Comparison of the number and distribution of hydrogen bonds around amino acid positions 89 and 227 within 5α-RD2 between WT and Hap1 (**E**), Hap2 (**F**), and Hap3 (**G**)

To investigate the structural impact of variants on 5α-RD2, Chai-1 and PyMOL were used for modelling and visualization, respectively. Interaction analyses of intramolecular hydrogen bonds were conducted, which were among residues within a 5 Å distance of two mutated sites (positions 89 and 227). The impact of the variants on the hydrogen bond network was localized, which was reflected mainly in changes in the number and distribution of hydrogen bonds around key residues. For WT 5α-RD2, in total, there were 14 hydrogen bonds, including those between L89 and G85 and between R227 and F223 (Fig. 4A), suggesting relatively strong interactions within 5α-RD2. In contrast, for Hap1: G-G-derived 5α-RD2, the number of hydrogen bonds (n = 14) was the same as that in the WT, and the hydrogen bond between V89 and G85 remained (Fig. 4A, B and E). However, the hydrogen bond between M157 and F153 was lost, whereas that between F229 and L226 gained (Fig. 4E). For Hap2: C-A-derived 5α-RD2, the number of hydrogen bonds (n = 16) was slightly greater than that in the WT (n = 14) (Fig. 4A, C, and F). However, notably, the key hydrogen bond between R227 and F223 was replaced by a hydrogen bond between Q227 and F223 (Fig. 4F). In the case of Hap3: G-A, the number of hydrogen bonds decreased (WT: 14 vs. Hap3: 13), and the hydrogen bond network was redistributed. The hydrogen bond between V89 and G85 was maintained at 2.9 Å, whereas the hydrogen bond distance between Q227 and F223 was 3.0 Å (Fig. 4A, D, and G). Hap3: G-A resulted in relatively large changes in the hydrogen bond network, ultimately affecting the overall stability of 5α-RD2.

### SASA analysis of the 3D structure composed of 5α-RD2 with NADPH and testosterone

Four ternary complex models were constructed, each consisting of 5α-RD2 (derived from WT, Hap1, Hap2, or Hap3), NADPH, and testosterone. The SASA was calculated for each of the ternary complex models. Compared with that of the WT, the SASA of the Hap1: G-G-based model increased by 33.03 Å^2^ (Table 3). This suggested that Hap1: G-G likely shortened the hydrophobic side chain of 5α-RD2, thereby fine-tuning the 5α-RD2’ conformation. In comparison, Hap2: C-A and Hap3: G-A only mildly increased the SASA by 2.45 Å^2^ and 10.72 Å^2^, respectively (Table 3). Since testosterone is a hydrophobic molecule, the larger the SASA is, the less likely testosterone is to be recruited near the enzyme; thus, Hap1: G-G may cause a greater decrease in 5α-RD2 enzymatic binding with testosterone than Hap2: C-A or Hap3: G-A.

**Table 3:** SASA of the ternary models composed of 5α-RD2, NADPH and testosterone

Group	SASA (Å^2^)	Change
WT: C-G (L89, R227)	25,700.67	–
Hap1: G-G (L89V)	25,733.70	+ 33.03
Hap2: C-A (R227Q)	25,703.12	+ 2.45
Hap3: G-A (L89V, R227Q)	25,711.39	+ 10.72

### Interaction analysis of 5α-RD2 with NADPH and testosterone

On the basis of the previously published covalent crystal structure of 5α-RD2, the active region of the enzyme for ligand binding was estimated. The NADPH interaction region might be within a 5 Å radius around position 227 of 5α-RD2, whereas testosterone might be located within a 5 Å radius around position 114 of 5α-RD2. Accordingly, ternary structure models were built.

**Fig. 5:**
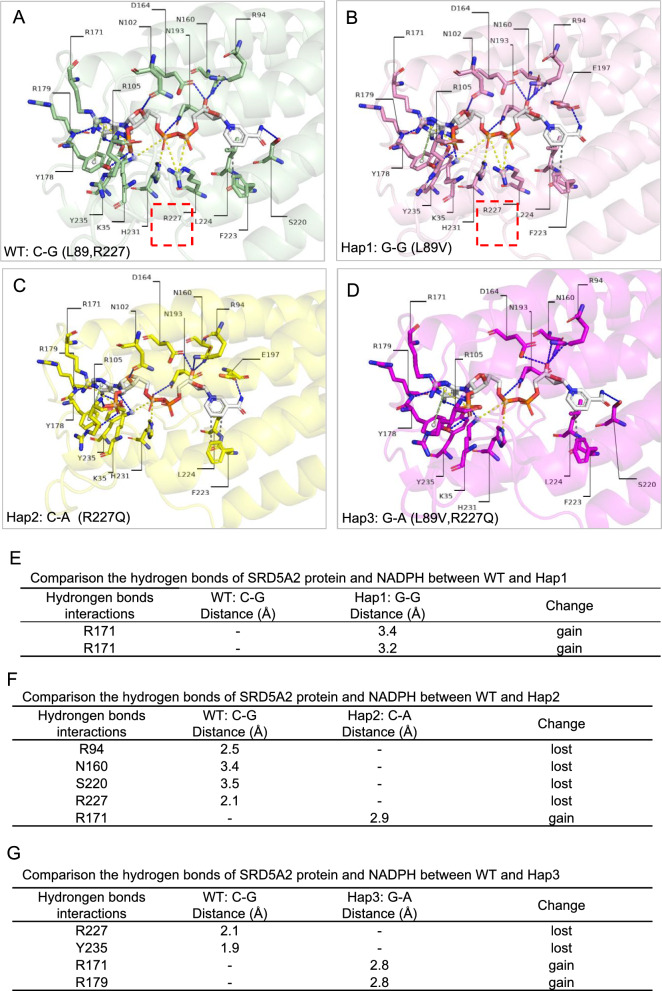
Interaction analysis of 5α-RD2 with NADPH. **A**–**D** Visualization of hydrogen bonds between NADPH and 5α-RD2 derived from WT: C-G (**A**), Hap1: G-G (**B**), Hap2: C-A (**C**), and Hap3: G-A (**D**), respectively. The NADPH is shown as a gray stick. The key residue R227 that binds NADPH is highlighted with a red box. **E**–**G** Comparison of hydrogen bond number and distribution of 5α-RD2 and NADPH for WT: C-G and Hap1: G-G (**E**), Hap2: C-A (**F**), and Hap3: G-A (**G**)

In the WT 5α-RD2 and NADPH model, several positively charged residues (K35, R94, R105, R171, R179, R227, etc.) formed salt bridges/hydrogen bonds with the phosphate group of NADPH (Fig. 5A), which were essential for maintaining high affinity between 5α-RD2 and NADPH. In addition, peripheral polar residues (N160, N193, Y235, etc.) provided supplementary hydrogen bonds, and hydrophobic residues (F223, L224, etc.) helped anchor the adenine ring (Fig. 5A). The hydrogen bonding interaction between the positively charged R227 and the phosphate group of NADPH was the most critical factor determining the strength of enzyme-cofactor binding. Compared with WT, Hap1: G-G retained R227 hydrogen bond interactions with NADPH, thus showing a relatively limited influence on 5α-RD2 binding with NADPH (Fig. 5B and E). However, Hap2: C-A significantly disrupted the original network of positive charge and hydrogen bond interactions (such as R227 and NADPH) associated with the phosphate group of NADPH, leading to the loss of key salt bridges and hydrogen bonds with NADPH, thus directly significantly weakening the binding stability of 5α-RD2 and NADPH (Fig. 5C and F). For Hap3: G-A, owing to arginine substituted by glutamine at position 227, the essential hydrogen bond between R227 and NADPH was lost; however, owing to the effect of leucine replacement by valine at position 89, the remaining hydrogen bond network was relatively complex (Fig. 5D and G).

Since testosterone primarily interacts with 5α-RD2 through hydrophobic residues (such as F219, F223, and some conserved hydrophobic sites) and a few hydrogen-bonding residues in both the WT and Hap1/2/3 models, positions 89 and 227 were closer to the NADPH-binding region than to the testosterone-binding region, their direct impact on the testosterone-binding site was relatively minor.

### *SRD5A2* Hap3: G-A affected 5α-RD2 activity kinetics with a hypomorphic effect

**Fig. 6:**
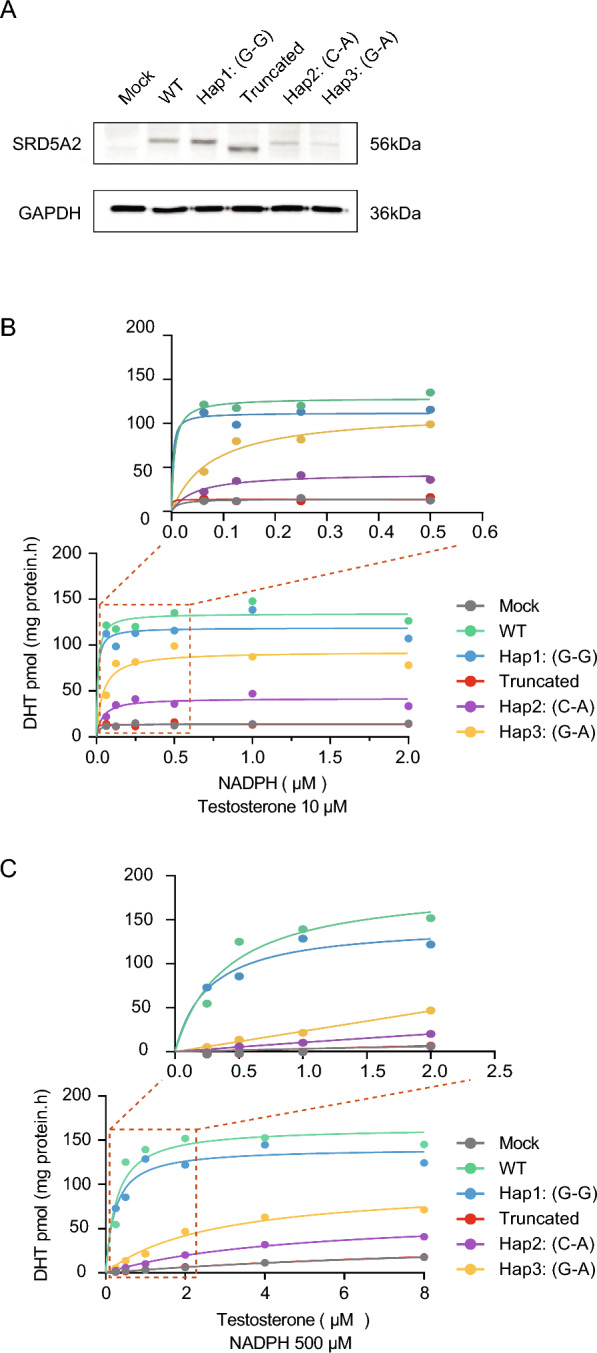
*SRD5A2* Hap3: G-A inhibited the catalytic efficiency of the enzymes. **A** Protein expression levels of SRD5A2 from all overexpression plasmids. The WB experiments were performed in triplicate. **B** Catalytic activity of 5α-RD2, measured by dihydrotestosterone (DHT) production, across a range of NADPH concentrations at a fixed testosterone concentration (10 μM). **C** Catalytic activity of 5α-RD2, measured by DHT production, across a range of testosterone concentrations at a fixed NADPH concentration (500 μM). The mock vector was an empty control vector expressing only GFP. The LC-MS/MS experiments were replicated three times

To validate the functional defects caused by the *SRD5A2* haplotypes, in vitro protein overexpression experiments were conducted. The empty vector plasmid expressing only GFP served as the mock control, while the WT and truncated mutant (c.679C > T, p.R227*) *SRD5A2* plasmids were used as positive and negative controls, respectively. WB results confirmed the validity of the transfection and overexpression of different haplotypes (Fig. 6A). As expected, LC–MS/MS analysis showed that, similar to the mock control, the truncated variant largely decreased DHT production when cultured with various concentrations of NADPH or testosterone (Fig. 6B–C). When testosterone was 10 μM, NADPH with different concentrations, compared to WT (Vmax = 134.28 ± 28.18 pmol/mg protein/min), the Vmax values of Hap1: G-G, Hap2: C-A and Hap3: G-A were 118.71 ± 26.08, 41.80 ± 6.49, and 92.70 ± 19.15 pmol/mg protein/min, respectively. The corresponding catalytic efficiencies decreased to 88.40%, 31.13%, and 69.03%, respectively (Fig. 6B). Similarly, when NADPH was at 500 μM, testosterone with different concentrations, compared to WT (Vmax = 164.23 ± 26.80 pmol/mg protein/min), the Vmax values of Hap1: G-G, Hap2: C-A and Hap3: G-A were 140.85 ± 25.90, 65.94 ± 26.22, and 100.31 ± 35.33 pmol/mg protein/min, respectively. The corresponding catalytic efficiencies decreased to 85.76%, 40.15%, and 61.08%, respectively (Fig. 6C). Collectively, the catalytic efficiencies of the enzymes encoded by Hap1: G-G, Hap2: C-A, and Hap3: G-A all ranked between those of the WT and the truncated variant. Notably, the enzyme encoded by Hap3: G-A exhibited an intermediate catalytic efficiency between that of Hap1: G-G and Hap2: C-A.

## Discussion

Individuals with DSD, for example, 5α-RD2 deficiency, exhibit high genetic and phenotypic heterogeneity, and the genotype–phenotype correlation remains unclear [34]. In this study, we identified a novel *SRD5A2* haplotype (Hap3: G-A) that may have a potential founder effect by characterizing population distribution and differentiation. 3D structure prediction and model construction suggested that Hap3: G-A may have a combined effect on the structure and interaction of 5α-RD2 with ligands, as supported by functional assays directly demonstrating its hypomorphic effect on enzyme activity.

The founder effect is the establishment of a new population from a small group of founding individuals with similar genetic characteristics, partially due to internal preferences resulting in geographical and cultural isolation [35]. To investigate the founder effect in individuals with 5α-RD2 or non-5α-RD2 deficiency DSD, the majority of studies have focused on examining a single variant [16, 36], even though few studies have performed haplotype analysis [10, 25]. For example, Zhu et al. performed haplotyping focusing on p.G203S but did not find a founder effect based on the frequency profile of the specific haplotype [10], probably due to the limited number of enrolled cases. Haplotyping is important for identifying disease-associated variants [37, 38], mapping recombination patterns [39], tracing ancestry [40–42], and offering valuable insights into the pathogenesis of diseases [26, 43, 44]. Our previous multicenter study reported that the unique prevalence of c.680G > A in the Chinese population suggested a putative founder effect of *SRD5A2* [24, 45]. On the basis of large-scale participants, including 216 cases and 2,794 controls, this study identified a *SRD5A2* haplotype in linkage (Hap3: G-A, represented by c.265C > G and c.680G > A), which was frequently found in individuals with 5α-RD2 deficiency but had a low frequency in in-house and public controls across multiple ethnogeographic groups. Globally, Hap3: G-A showed different distributions between southern Chinese populations and other populations, probably resulting from a potential founder effect, which might be due to geographical isolation from a large coefficient of inbreeding [14, 46, 47]. This finding was further supported by the high population differentiation between southern Chinese individuals and populations of African, European or American ancestry, in contrast to the low differentiation between southern Chinese individuals and populations of East or South Asian ancestry. The distinctive distribution of Hap3: G-A between North China and South China seems to indicate further splitting of the Chinese population into subgroups with divergent genetic backgrounds and multiethnic origins. These findings underscore the importance of considering regional genetic variations when studying the epidemiology and clinical presentation of DSD. The existence of the prevalent haplotype might indicate a shared ancestry among cases. To the best of our knowledge, this is the first study investigating *SRD5A2* with a founder effect from the perspective of haplotyping, thus providing new insights into the diverse genetic background of 5α-RD2 deficiency.

Several molecular pathways, including steroid hormone biosynthesis pathway and others, are involved in the sex development process. These signalling pathways form a complex regulatory network, affecting the presence and variability of phenotypes in DSDs. Our previous analysis of the phenotype-genotype correlation of *SRD5A2* in individuals with 5α-RD2 deficiency found that c.680G > A predominated in cases with normal meatus or distal hypospadias compared with those with proximal hypospadias [24]. In addition, c.265C > G was traditionally considered a benign variant because of its high frequency in the normal population; thus, its correlation with phenotype has rarely been investigated [48]. Interestingly, we found that Hap3: G-A of *SRD5A2* was prevalent in individuals with 5α-RD2 deficiency and exhibited a trend of higher frequency in non-5α-RD2 deficiency DSD cases without known DSD-related gene variants than in those with known DSD-related gene variants, suggesting that Hap3: G-A may play an important role in the regulatory network underlying DSD. Variants in common DSD-related genes, such as AR, have been detected in combination with additional variants in other genes in DSD individuals with a broad range of phenotypes, implying a role of oligogenic inheritance in DSD [49]. The effects of some variants of DSD-related genes might be minor or moderate in affecting disease pathogenesis; however, these variants can accumulate with Hap3: G-A of *SRD5A2* or other gene variants to perturb the regulatory network of sex development, thus contributing to disease risk. An analysis of the phenotype spectrum demonstrated that the majority of homozygotes of Hap3: G-A presented microphallus, and nearly half of them manifested isolated microphallus. It was expected that homozygotes of Hap3: G-A might exhibit relatively mild phenotypes. These findings provide a novel perspective for elucidating the genetic complexity and phenotypic variability observed in DSD individuals.

Structural prediction analysis provides theoretical insights for understanding the function of *SRD5A2* haplotypes. Intramolecular hydrogen bonds primarily stabilize tertiary structures [50], whereas intermolecular hydrogen bonds are essential for molecular recognition, signal transduction, and enzymatic catalytic activity [51, 52]. Hap1: G-G mainly increased the SASA of the complex but mildly influenced the stability of the enzyme and its interactions with the substrate testosterone and cofactor NADPH, which is consistent with its mild effect on the catalytic activity observed in kinetic assays. In contrast, Hap2: C-A was predicted to disrupt 5α-RD2 binding with NADPH by reducing the number of hydrogen bonds, which is consistent with its largely decreased catalytic activity. Hap3: G-A lost significant interaction with NADPH because of R227Q, whereas L89V increased 5α-RD2 stability locally by shortening the side chain, which compensated for the disruption effect of R227Q. Overall, Hap3: G-A seemed to have a combined effect on the structure and interaction of 5α-RD2, rather than simply additive effects of its constituent variants. Further kinetic assays confirmed that the catalytic efficiencies of the enzymes encoded by Hap1: G-G, Hap2: C-A, and Hap3: G-A all ranked between those of the WT and the truncated variant, suggesting a hypomorphic effect of these haplotypes. Notably, the enzyme encoded by Hap3: G-A exhibited an intermediate catalytic efficiency between that of Hap1: G-G and Hap2: C-A, probably attributable to the differential effects of these haplotypes on the binding of 5α-RD2 with testosterone or NADPH.

To further elucidate the associations between specific haplotypes and clinical phenotypes, expanding the cohort by recruiting more individuals with 5α-RD2 deficiency and other DSDs would be beneficial. Future research should focus on validating structural changes via experiments or molecular dynamics simulations to gain a deeper understanding of kinetic properties and functional mechanisms. In addition to in vitro experiments, in vivo validation in animal models to elucidate the role of specific haplotypes, as well as their interactions with DSD-related regulatory networks, might be considered.

### Perspectives and significance

This study identified a novel hypomorphic *SRD5A2* haplotype with a potential founder effect, which was composed of common variants. These findings provide valuable insights for understanding complex genotype–phenotype correlations through haplotyping of specific genes, facilitating genetic counselling, early intervention and clinical management of individuals with 5α-RD2 deficiency or even other DSDs. Notably, in future clinical practice, analyses of genetic factors underlying 5α-RD2 deficiency and other DSDs should shift from focusing solely on individual variants to accounting for the interactions and combined effects of multiple variants, particularly those in linkage, potentially offering new clues for therapeutic targets for affected individuals.

## Conclusions

This study identified a novel *SRD5A2* haplotype with a potential founder effect in individuals with 5α-RD2 deficiency from South China, which was composed of altered alleles from relatively common variants, c.265C > G and c.680G > A. Structural prediction indicated molecular changes underlying the potential role of this haplotype in gene dysfunction, which was supported by functional assays directly demonstrating its hypomorphic effect on enzyme activity. These findings provide valuable insights for understanding genotype–phenotype correlations, genetic counselling, early intervention and clinical management of individuals with 5α-RD2 deficiency or even other DSDs.

## Supplementary Information

Additional file 1.

Additional file 2.

Additional file 3.

Additional file 4.

Additional file 5.

Additional file 6.

Additional file 7.

Additional file 8.

## Data Availability

The data that support the findings of this study are obtained in the article or can be made available upon request.

## References

[1] Bennecke E, Thyen U, Grüters A, Lux A, Köhler B. Health-related quality of life and psychological well-being in adults with differences/disorders of sex development. Clin Endocrinol (Oxf). 2017;86(4):634–43. 28005277 10.1111/cen.13296

[2] Ono M, Harley VR. Disorders of sex development: new genes, new concepts. Nat Rev Endocrinol. 2013;9(2):79–91. 23296159 10.1038/nrendo.2012.235

[3] Wisniewski AB, Batista RL, Costa EMF, Finlayson C, Sircili MHP, Dénes FT, et al. Management of 46,XY differences/disorders of sex development (DSD) throughout life. Endocr Rev. 2019;40(6):1547–72. 31365064 10.1210/er.2019-00049

[4] Cools M, Nordenström A, Robeva R, Hall J, Westerveld P, Flück C, et al. Caring for individuals with a difference of sex development (DSD): a consensus statement. Nat Rev Endocrinol. 2018;14(7):415–29. 29769693 10.1038/s41574-018-0010-8 PMC7136158

[5] Yu BQ, Liu ZX, Gao YJ, Wang X, Mao JF, Nie M, et al. Prevalence of gene mutations in a Chinese 46,XY disorders of sex development cohort detected by targeted next-generation sequencing. Asian J Androl. 2021;23(1):69–73. 32985417 10.4103/aja.aja_36_20 PMC7831832

[6] Chen H, Chen G, Li F, Huang Y, Zhu L, Zhao Y, et al. Application and insights of targeted next-generation sequencing in a large cohort of 46,XY disorders of sex development in Chinese. Biol Sex Differ. 2024;15(1):73. 39285472 10.1186/s13293-024-00648-6 PMC11403886

[7] Xiao Q, Wang L, Supekar S, Shen T, Liu H, Ye F, et al. Structure of human steroid 5α-reductase 2 with the anti-androgen drug finasteride. Nat Commun. 2020;11(1):5430. 33110062 10.1038/s41467-020-19249-z PMC7591894

[8] Imperato-McGinley J, Guerrero L, Gautier T, Peterson RE. Steroid 5α-reductase deficiency in man: an inherited form of male pseudohermaphroditism. Science. 1974;186(4170):1213–5. 4432067 10.1126/science.186.4170.1213

[9] Mendonca BB, Batista RL, Domenice S, Costa EM, Arnhold IJ, Russell DW, et al. Reprint of “Steroid 5α-reductase 2 deficiency.” J Steroid Biochem Mol Biol. 2017;165(Pt A):95–100. 27842977 10.1016/j.jsbmb.2016.11.006

[10] Zhu H, Liu W, Han B, Fan M, Zhao S, Wang H, et al. Phenotypic and molecular characteristics in eleven Chinese patients with 5α-reductase Type 2 deficiency. Clin Endocrinol (Oxf). 2014;81(5):711–20. 24665940 10.1111/cen.12456

[11] Reyes AP, León NY, Frost ER, Harley VR. Genetic control of typical and atypical sex development. Nat Rev Urol. 2023;20(7):434–51. 37020056 10.1038/s41585-023-00754-x

[12] Fagerberg L, Hallström BM, Oksvold P, Kampf C, Djureinovic D, Odeberg J, et al. Analysis of the human tissue-specific expression by genome-wide integration of transcriptomics and antibody-based proteomics. Mol Cell Proteomics. 2014;13(2):397–406. 24309898 10.1074/mcp.M113.035600 PMC3916642

[13] Khorashad BS, Gardner M, Lee PA, Kogan BA, Sandberg DE. Recommendations for 46,XY disorders/differences of sex development across two decades: insights from North American pediatric endocrinologists and urologists. Arch Sex Behav. 2024;53(8):2939–56. 39039338 10.1007/s10508-024-02942-1 PMC11335971

[14] Imperato-McGinley J, Zhu YS. Androgens and male physiology the syndrome of 5α-reductase-2 deficiency. Mol Cell Endocrinol. 2002;198(1–2):51–9. 12573814 10.1016/s0303-7207(02)00368-4

[15] Imperato-McGinley J, Guerrero L, Gautier T, German JL, Peterson RE. Steroid 5alpha-reductase deficiency in man. An inherited form of male pseudohermaphroditism. Birth Defects Orig Artic Ser. 1975;11(4):91–103. 1156691

[16] Zhang M, Yang J, Zhang H, Ning G, Li X, Sun S. A novel SRD5A2 mutation with loss of function identified in Chinese patients with hypospadias. Horm Res Paediatr. 2011;76(1):44–9. 21540559 10.1159/000324692

[17] Zhang W, Yu B, Luo W, Sun B, Zhang X, Wang X, et al. In vitro functional study of fifteen SRD5A2 variants found in Chinese patients and the relation between the SRD5A2 genotypes and phenotypes. J Steroid Biochem Mol Biol. 2023;235:106421. 37918676 10.1016/j.jsbmb.2023.106421

[18] Landrum MJ, Chitipiralla S, Kaur K, Brown G, Chen C, Hart J, et al. ClinVar: updates to support classifications of both germline and somatic variants. Nucleic Acids Res. 2025;53(D1):D1313–21. 39578691 10.1093/nar/gkae1090 PMC11701624

[19] Ramos L, Vilchis F, Chávez B, Mares L. Mutational analysis of SRD5A2: from gene to functional kinetics in individuals with steroid 5α-reductase 2 deficiency. J Steroid Biochem Mol Biol. 2020;200:105691. 32380235 10.1016/j.jsbmb.2020.105691

[20] Makridakis N, Ross RK, Pike MC, Chang L, Stanczyk FZ, Kolonel LN, et al. A prevalent missense substitution that modulates activity of prostatic steroid 5α-reductase. Cancer Res. 1997;57(6):1020–2. 9067262

[21] Makridakis NM, di Salle E, Reichardt JK. Biochemical and pharmacogenetic dissection of human steroid 5 alpha-reductase type II. Pharmacogenetics. 2000;10(5):407–13. 10898110 10.1097/00008571-200007000-00004

[22] Karczewski KJ, Francioli LC, Tiao G, Cummings BB, Alfoldi J, Wang Q, et al. The mutational constraint spectrum quantified from variation in 141,456 humans. Nature. 2020;581(7809):434–43. 32461654 10.1038/s41586-020-2308-7 PMC7334197

[23] Gui T, Yao F, Yang X, Wang X, Nie M, Wu X, et al. Genotype-phenotype correlation analysis and identification of a novel SRD5A2 mutation in four unrelated Chinese patients with 5α-reductase deficiency. Int J Gen Med. 2022;15:6633–43. 36016984 10.2147/IJGM.S377675 PMC9395993

[24] Gui B, Song Y, Su Z, Luo FH, Chen L, Wang X, et al. New insights into 5α-reductase type 2 deficiency based on a multi-centre study: regional distribution and genotype-phenotype profiling of SRD5A2 in 190 Chinese patients. J Med Genet. 2019;56(10):685–92. 31186340 10.1136/jmedgenet-2018-105915

[25] Han B, Cheng T, Zhu H, Yu J, Zhu WJ, Song HD, et al. Genetic analysis of 25 patients with 5α-reductase deficiency in Chinese population. Biomed Res Int. 2020;2020:1789514. 32596280 10.1155/2020/1789514 PMC7301183

[26] Wu N, Ming X, Xiao J, Wu Z, Chen X, Shinawi M, et al. TBX6 null variants and a common hypomorphic allele in congenital scoliosis. N Engl J Med. 2015;372(4):341–50. 25564734 10.1056/NEJMoa1406829 PMC4326244

[27] Auton A, Brooks LD, Durbin RM, Garrison EP, Kang HM, Korbel JO, et al. A global reference for human genetic variation. Nature. 2015;526(7571):68–74. 26432245 10.1038/nature15393 PMC4750478

[28] Li H, Durbin R. Fast and accurate long-read alignment with Burrows-Wheeler transform. Bioinformatics. 2010;26(5):589–95. 20080505 10.1093/bioinformatics/btp698 PMC2828108

[29] Patel RK, Jain M. NGS QC toolkit: a toolkit for quality control of next generation sequencing data. PLoS ONE. 2012;7(2):e30619. 22312429 10.1371/journal.pone.0030619 PMC3270013

[30] McKenna A, Hanna M, Banks E, Sivachenko A, Cibulskis K, Kernytsky A, et al. The genome analysis toolkit: a MapReduce framework for analyzing next-generation DNA sequencing data. Genome Res. 2010;20(9):1297–303. 20644199 10.1101/gr.107524.110 PMC2928508

[31] Purcell S, Neale B, Todd-Brown K, Thomas L, Ferreira MA, Bender D, et al. PLINK: a tool set for whole-genome association and population-based linkage analyses. Am J Hum Genet. 2007;81(3):559–75. 17701901 10.1086/519795 PMC1950838

[32] Chai Discovery JB, Dent J, McPartlon M, Meier J, Reis V, Rogozhnikov A, Wu K. Chai-1: Decoding the molecular interactions of life. 2024. Preprint at https://www.biorxiv.org/content/10.1101/2024.10.10.615955v2.full.pdf.

[33] Seeliger D, de Groot BL. Ligand docking and binding site analysis with PyMOL and Autodock/Vina. J Comput Aided Mol Des. 2010;24(5):417–22. 20401516 10.1007/s10822-010-9352-6 PMC2881210

[34] Shabir I, Khurana ML, Joseph AA, Eunice M, Mehta M, Ammini AC. Phenotype, genotype and gender identity in a large cohort of patients from India with 5α-reductase 2 deficiency. Andrology. 2015;3(6):1132–9. 26453174 10.1111/andr.12108

[35] Marafi D. Founder mutations and rare disease in the Arab world. Dis Model Mech. 2024;17(6):dmm050715. 38922202 10.1242/dmm.050715 PMC11225585

[36] Akiba K, Aso K, Hasegawa Y, Fukami M. Genome analyses and androgen quantification for an infant with 5α-reductase type 2 deficiency. J Pediatr Endocrinol Metab. 2021;34(9):1191–5. 34162032 10.1515/jpem-2020-0678

[37] Wang C, Dai J, Qin N, Fan J, Ma H, Chen C, et al. Analyses of rare predisposing variants of lung cancer in 6,004 whole genomes in Chinese. Cancer Cell. 2022;40(10):1223–39. 36113475 10.1016/j.ccell.2022.08.013

[38] Camp NJ, Lin WY, Bigelow A, Burghel GJ, Mosbruger TL, Parry MA, et al. Discordant haplotype sequencing identifies functional variants at the 2q33 breast cancer risk locus. Cancer Res. 2016;76(7):1916–25. 26795348 10.1158/0008-5472.CAN-15-1629 PMC4873429

[39] Glusman G, Cox HC, Roach JC. Whole-genome haplotyping approaches and genomic medicine. Genome Med. 2014;6(9):73. 25473435 10.1186/s13073-014-0073-7 PMC4254418

[40] Fournier R, Tsangalidou Z, Reich D, Palamara PF. Haplotype-based inference of recent effective population size in modern and ancient DNA samples. Nat Commun. 2023;14(1):7945. 38040695 10.1038/s41467-023-43522-6 PMC10692198

[41] Bredemeyer KR, Hillier L, Harris AJ, Hughes GM, Foley NM, Lawless C, et al. Single-haplotype comparative genomics provides insights into lineage-specific structural variation during cat evolution. Nat Genet. 2023;55(11):1953–63. 37919451 10.1038/s41588-023-01548-y PMC10845050

[42] Yang J, Xue H, Li Z, Zhang Y, Shi T, He X, et al. Haplotype-resolved genome assembly provides insights into the evolution of S-locus supergene in distylous *Nymphoides indica*. New Phytol. 2023;240(5):2058–71. 37717220 10.1111/nph.19264

[43] Dai J, Lv J, Zhu M, Wang Y, Qin N, Ma H, et al. Identification of risk loci and a polygenic risk score for lung cancer: a large-scale prospective cohort study in Chinese populations. Lancet Respir Med. 2019;7(10):881–91. 31326317 10.1016/S2213-2600(19)30144-4 PMC7015703

[44] Fu X, Li S, Zhao Z, Kong L, Zhu J, Li H, et al. Haplotype-based noninvasive prenatal diagnosis of methylmalonic acidemia and the discovery of a recurrent pathogenic haplotype associated with c.609G>A. Prenat Diagn. 2023;43(12):1544–55. 37957774 10.1002/pd.6458

[45] Fan L, Song Y, Polak M, Li L, Ren X, Zhang B, et al. Clinical characteristics and genotype-phenotype correlations of 130 Chinese children in a high-homogeneity single-center cohort with 5α-reductase 2 deficiency. Mol Genet Genomic Med. 2020;8(10):e1431. 32713132 10.1002/mgg3.1431 PMC7549558

[46] Cheng J, Lin R, Zhang W, Liu G, Sheng H, Li X, et al. Phenotype and molecular characteristics in 45 Chinese children with 5α-reductase type 2 deficiency from South China. Clin Endocrinol (Oxf). 2015;83(4):518–26. 25899528 10.1111/cen.12799

[47] Vilchis F, Ramos L, Méndez JP, Benavides S, Canto P, Chávez B. Molecular analysis of the SRD5A2 in 46,XY subjects with incomplete virilization: the P212R substitution of the steroid 5α-reductase 2 may constitute an ancestral founder mutation in Mexican patients. J Androl. 2010;31(4):358–64. 20019388 10.2164/jandrol.109.009407

[48] Dalili S, Rabbani B, Hassanzadeh Rad A, Koohmanaee S, Mahdieh N. A novel pathogenic variant of SRD5A2 in an Iranian psuedohermaphrodite male. Clin Case Rep. 2020;8(10):1947–51. 33088526 10.1002/ccr3.3028 PMC7562857

[49] Kouri C, Sommer G, Flück CE. Oligogenic causes of human differences of sex development: facing the challenge of genetic complexity. Horm Res Paediatr. 2023;96(2):169–79. 34537773 10.1159/000519691

[50] Li Z, Li H, Zhao L, Liu X, Wan C. Understanding the role of cations and hydrogen bonds on the stability of aerobic granules from the perspective of the aggregation and adhesion behavior of extracellular polymeric substances. Sci Total Environ. 2021;795:148659. 34237538 10.1016/j.scitotenv.2021.148659

[51] Schiebel J, Gaspari R, Wulsdorf T, Ngo K, Sohn C, Schrader TE, et al. Intriguing role of water in protein-ligand binding studied by neutron crystallography on trypsin complexes. Nat Commun. 2018;9(1):3559. 30177695 10.1038/s41467-018-05769-2 PMC6120877

[52] Pedersen SW, Pedersen SB, Anker L, Hultqvist G, Kristensen AS, Jemth P, et al. Probing backbone hydrogen bonding in PDZ/ligand interactions by protein amide-to-ester mutations. Nat Commun. 2014;5:3215. 24477114 10.1038/ncomms4215

